# Supplementary material for: Treatment with a GSK-3β/HDAC Dual Inhibitor Restores Neuronal Survival and Maturation in an In Vitro and In Vivo Model of CDKL5 Deficiency Disorder
Source: Int J Mol Sci. 2021 May 31;22(11):5950. doi: 10.3390/ijms22115950 (PMC8198396; doi:10.3390/ijms22115950)
Supplement: Supplementary file 1 [file ijms-22-05950-s001.zip › ijms-1212824-supplementary.pdf]

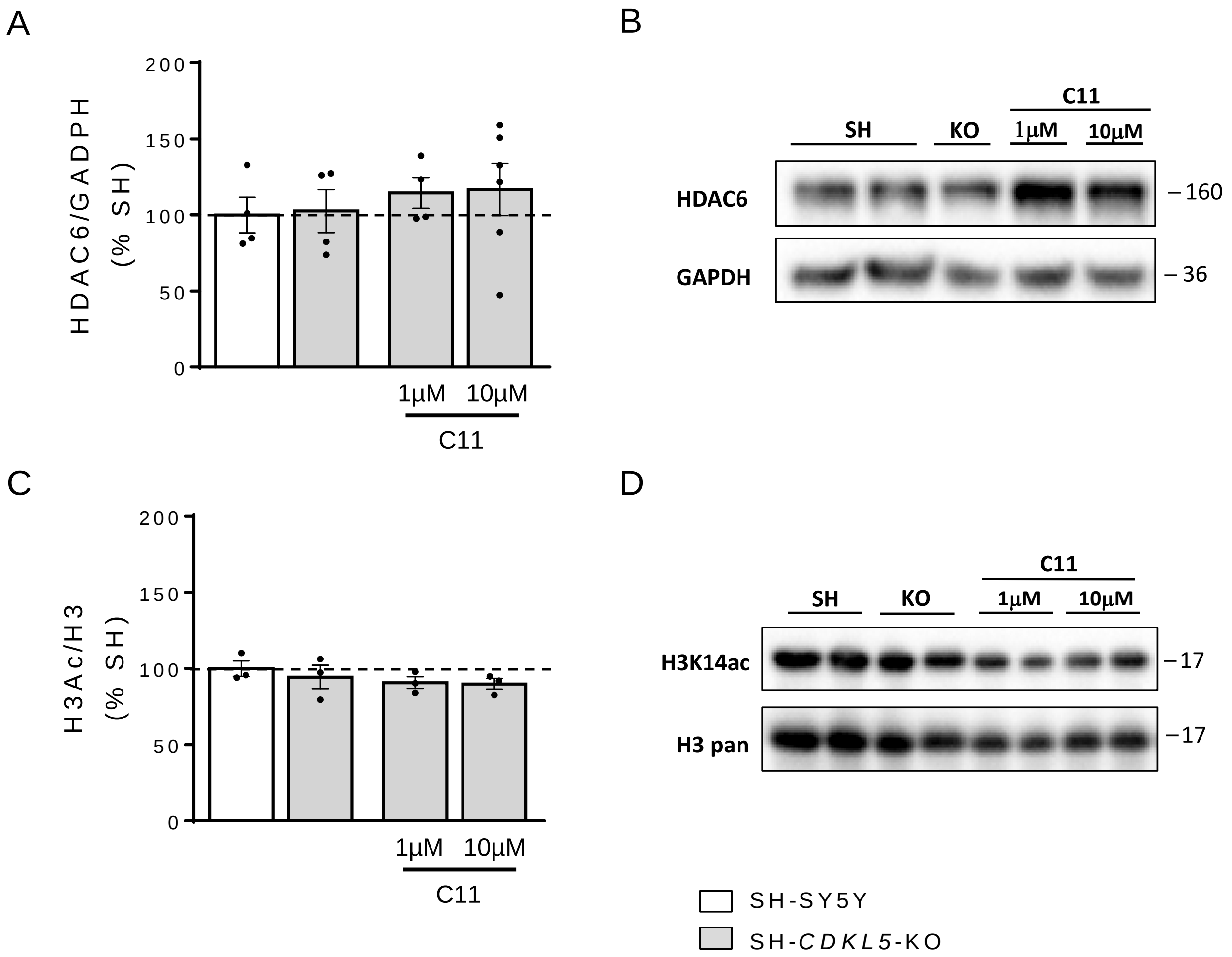

Figure S1. Effect of treatment with C11 on HDAC6 and acetylated H3 Histone levels in SH-*CDKL5*-KO cells. (A-D) Western blot analysis of HDAC6 and acetylated H3 Histone (H3Ac) levels in protein extracts from parental SH-SY5Y cells (SH), SH-*CDKL5*-KO cells (SKO) and SH-*CDKL5*-KO cells treated with C11 (1 µM or 10 µM) for 24 h. Immunoblots (B,D) are examples from two biological replicates of each experimental condition. Histograms on the left show HDAC6 protein levels normalized to GAPDH and H3Ac protein levels normalized to corresponding total protein levels (H3). Data are expressed as a percentage of parental cells. Values are represented as means SE.

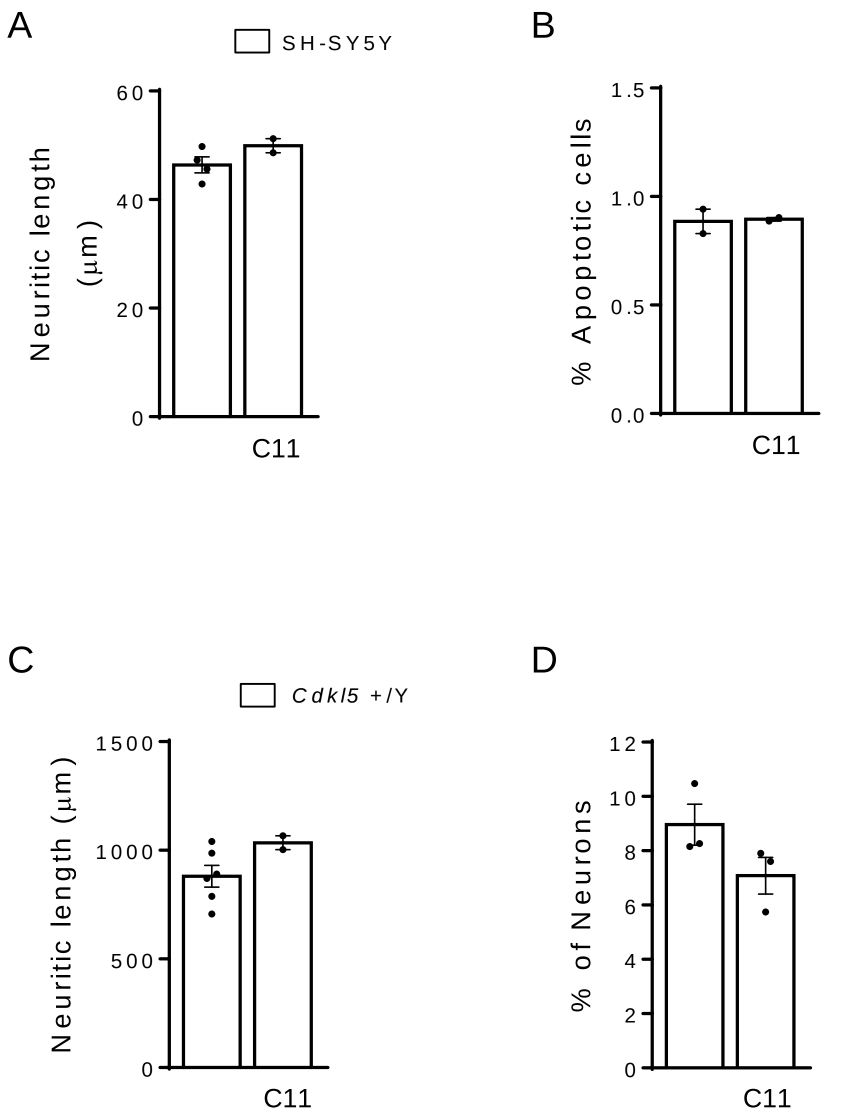

Figure S2. Effect of treatment with C11 on cell differentiation, proliferation and survival in SH-CDKL5 cells and wild-type hippocampal neurons.

(A) Quantification of neurite outgrowth of SH-SY5Y cells treated daily with retinoic acid (RA; 10 μM, n = 4) for 5 days. Cells were treated with C11 (10 μM, n = 2) every 2 days during retinoic acid differentiation. (B) Percentage of pyknotic nuclei in proliferating SH-SY5Y cells (n = 2); cells were treated with C11 (10 μM, n = 2) for 24 h. (C) Quantification of the total length of MAP2-positive cells from 10-day differentiated (DIV10) hippocampal neurons of *Cdkl5* +/Y mice. On day 2 post-plating (DIV2) hippocampal cultures were treated with vehicle (0.1% DMSO in PBS; *Cdkl5* +/Y; n = 6) or C11 (10 μM; n = 2), which was then administered on alternate days throughout the entire differentiation period. (D) Quantitative analysis of the number of MAP2-positive cells in vehicle-treated (n = 3) and C11-treated (C11; n = 3) hippocampal cultures from *Cdkl5* +/Y mice. Values are represented as mean ± SE.

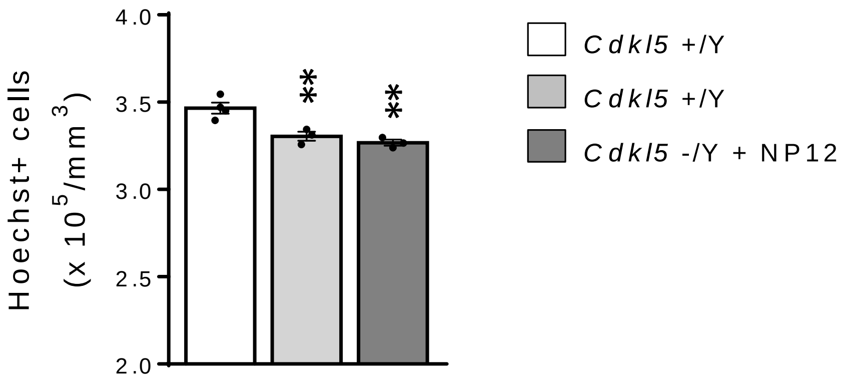

Figure S3. Effect of treatment with NP12 on neuronal survival in the hippocampus of *Cdkl5* -/Y mice.

(A) Quantification of Hoechst-positive cells in CA1 layer of hippocampal sections from vehicle-treated (*Cdkl5* +/Y; n = 4, *Cdkl*5 -/Y; n = 5) and NP12-treated (*Cdkl*5 -/Y + NP12; n = 4) mice, derived from animals used in [26]. Briefly, starting from 3 weeks of age, *Cdkl5* +/Y and *Cdkl5* -Y mice were treated either with vehicle (corn oil) or with NP12 (20 mg/kg), administered by subcutaneous injection every other day for 20 days. Values are represented as means ±SE. **p<0.01 (Fisher's LSD test after one-way ANOVA).

Supplementary Table 1

Antibody against	Description	Diluition	Product nr and Manufacturer	Use *
gamma H2A.X (phospho S139)	Rabbit polyclonal	1:1000	ab11174, Abcam	WB
phospho-AKT- Ser473	Rabbit polyclonal	1:1000	4060, Cell Signaling Technology	WB
AKT	Rabbit polyclonal	1:1000	4061, Cell Signaling Technology	WB
phospho-GSK-3β (Ser9) (D85E12) XP	Rabbit polyclonal	1:1000	5558, Cell Signaling Technology	WB
GSK-3β (27C10)	Rabbit polyclonal	1:1000	9315, Cell Signaling Technology	WB
Tubulin, Acetylated	Mouse monoclonal	1:1000	T6793, Sigma-Aldrich	WB
α-Tubulin	Mouse monoclonal	1:1000	T5168, Sigma-Aldrich	WB
phospho-CRMP-2 (Thr514)	Rabbit polyclonal	1:1000	9397, Cell Signaling Technology	WB
CRMP-2	Rabbit polyclonal	1:1000	9393, Cell Signaling Technology	WB
H3K9/14ac	Rabbit polyclonal	1:500	C15410005, Diagenode	WB
Histone H3, CT, pan	Rabbit monoclonal	1:1000	05-928, Merck Millipore, Burlington, MA, USA	WB
CDKL5	Sheep polyclonal	1:1000	SA145, MRC PPU reagent and service, University of Dundee, UK	WB
HDAC6	Rabbit polyclonal	1:1001	E-AB-63502, Elabscience	WB
GAPDH	Rabbit polyclonal	1:5000	G9545, Sigma-Aldrich	WB
NeuN , clone A60	Mouse monoclonal	1:250	MAB377, Merck Millipore, Burlington, MA, USA	IF
IBA1 Antibody (AIF-1)	Rabbit polyclonal	1:300	PA5-21274, ThermoFisher Scientific	IF
PSD95	Mouse monoclonal	1:1000	ab2723, Abcam	IF
Microtubule-Associated Protein 2 (MAP2)	Rabbit polyclonal	1:100	AB5622, Merck Millipore, Burlington, MA, USA	IF
		**Secondary antibodies**		
**Antibody**	**Conjugate**	**Diluition**	**Product nr and Manufacturer**	**Use***
Goat Anti-Rabbit IgG	HRP	1:5000	111-035-003, Jackson Im- munoResearch Laboratories, Inc.	WB
Donkey Anti-Sheep IgG	HRP	1:5000	713-005-147, Jackson Im- munoResearch Laboratories, Inc.	WB
Goat Anti-Mouse IgG	HRP	1:5000	115-005-003, Jackson Im- munoResearch Laboratories, Inc.	WB
Goat Anti-Mouse IgG	Cy3	1:200	115-165-062, Jackson Im- munoResearch Laboratories, Inc.	IF
Donkey Anti-Rabbit IgG	Cy3	1:200	711-165-152, Jackson Im- munoResearch Laboratories, Inc.	IF

*WB, western blot; IF, immunofluorescent staining
